# Concentrated Growth Factors for Managing a Nonvital Maxillary Central Incisor With an Open Apex

**DOI:** 10.7759/cureus.69280

**Published:** 2024-09-12

**Authors:** Shakila Mahesh, Alpa Gupta, Bandana Panda

**Affiliations:** 1 Department of Microbiology, Manav Rachna Dental College, Faridabad, IND; 2 Department of Dentistry, Manav Rachna International Institute of Research and Studies, Faridabad, IND; 3 Department of Conservative Dentistry and Endodontics, Kalinga Institute of Medical Sciences, Bhubaneswar, IND

**Keywords:** apexification, biomedical scaffold, concentrated growth factor, open apex management, platelet-rich fibrin, platelet-rich plasmin

## Abstract

Apexification procedure on a necrotic, infected tooth with an open apex is achievable if the canal is adequately disinfected. This case report aims to add an endodontic case to the body of knowledge currently available on the application of concentrated growth factor (CGF). A 24-year-old man with a history of a fall 15 years earlier developed apical periodontitis and pulpal necrosis in his maxillary central incisor with an open apex. Following the preparation of the access cavity, a solution containing 20 mL of sodium hypochlorite solution (5.25%) and 10 mL of 0.2% chlorhexidine was used to irrigate the canal successfully, and paper points were used to dry it. The canal was covered with a calcium hydroxide dressing for 10 days. The patient's right antecubital vein yielded 10 mL of whole blood, from which CGF was made. After the removal of the intracanal dressing, the CGF was placed into the canal to act as apical matrix to stabilize the mineral trioxide aggregate. The canal underwent composite restoration after being obturated. One year later, the clinical examination showed that the tests for palpation and percussion were negative. Both the electric and cold pulp tests yielded positive results for the tooth. Regression of the periapical lesion was seen on radiographic evaluation. Our case report's findings lead us to the conclusion that, in certain circumstances, complete canal disinfection can be used to treat necrotic, infected immature teeth, and that CGF might make an excellent scaffold matrix for treating open apex cases.

## Introduction

The continual development of the dentition is consistently exposed to various mechanical or biological challenges related to injury, decay, and various developmental irregularities [1]. Extensive research has been conducted to address the treatment of nonvital newly erupted teeth with incomplete root apex formation to restore the dentin-pulp complex [2]. Due to the lack of blood supply in nonvital teeth, the root fails to grow and becomes prone to fractures over time. Based on the apical foramen width and root length, Cvek in 1992 classified root development into five stages. Teeth with widely divergent apical openings were categorized into less than one-fifth, one-fifth, and two-thirds of apical closure, and, finally, those with closed apical foramen and fully developed roots. [3]. The strategic application of Ca(OH)_2_ gathered international recognition and was considered the preferred material for the process of apexification prior to the introduction of mineral trioxide aggregate (MTA) [4]. Recent efforts have focused on using platelet concentrates like platelet-rich plasma (PRP) and platelet-rich fibrin (PRF) as scaffold matrices for apexification procedures [5]. PRP has disadvantages with regard to handling, expense, intricate centrifugation, and purifying procedures, whereas PRF is simpler and more cost-effective, eliminating the need for bovine thrombin and anticoagulants [6]. Apart from traditional platelet concentrates, concentrated growth factor (CGF) has been suggested as an optimal biomaterial since it releases growth factors well known to encourage angiogenesis and tissue remodeling, as well as cell migration and proliferation. It also chemotactically acts on inflammatory cells [7]. We used the checklist provided in the Preferred Reporting Items for Case Reports in Endodontics 2020 guidelines to describe the case [8].

## Case presentation

**Figure 1:**
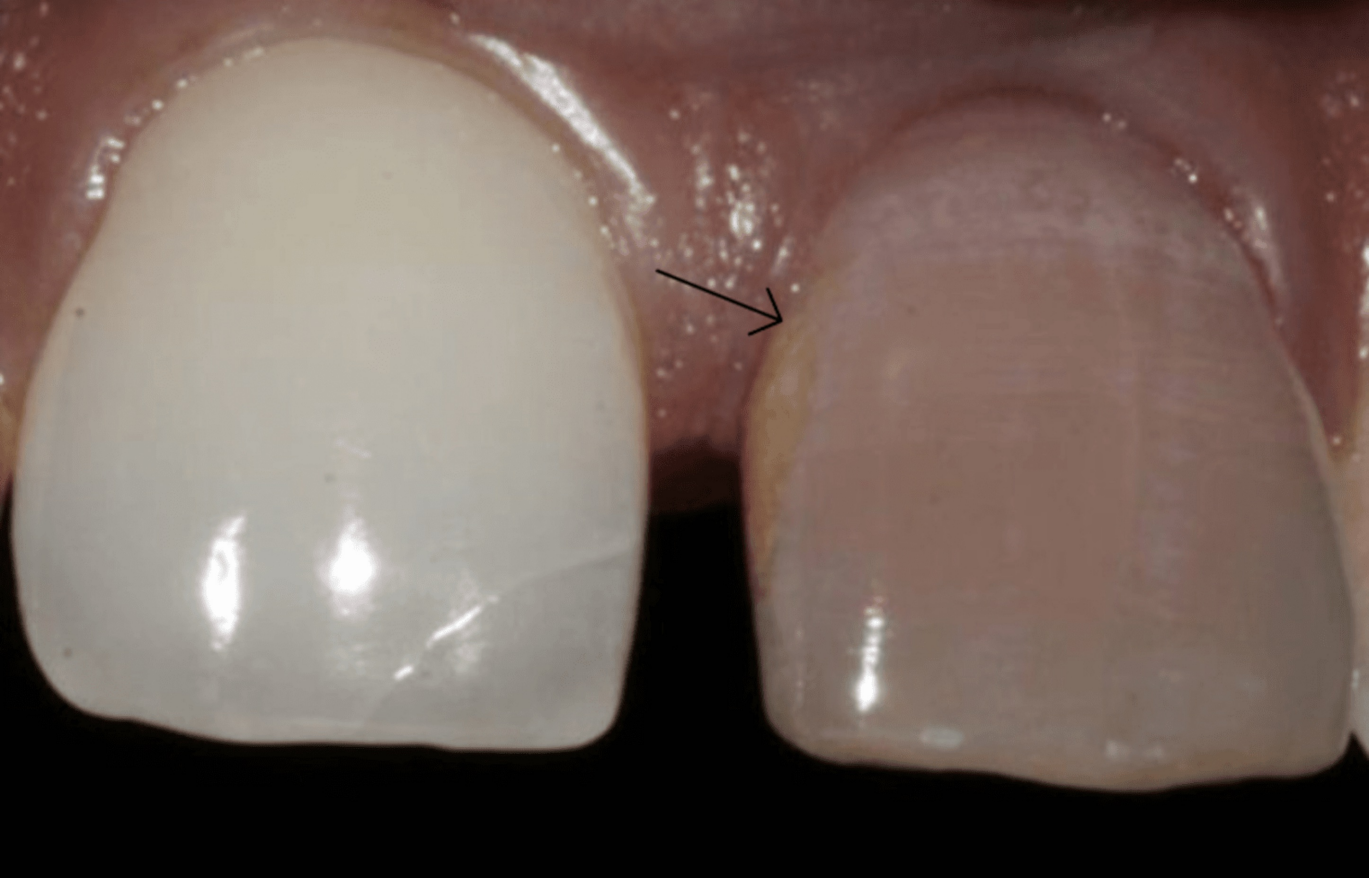
Preoperative intraoral clinical photograph showing the discolored anterior tooth 21 (black arrow)

A 24-year-old male patient visited the Conservative Dentistry and Endodontics specialty clinic, citing pain and discoloration around his upper front teeth. His medical background was unknown. Fifteen years ago, he had a sudden fall from the staircase. He fell face down, and the first impact was on the paraoral area, which was associated with severe discomfort while biting. A clinical examination of the mouth revealed discolored teeth that responded to pressure and percussion (Figure 1).

**Figure 2:**
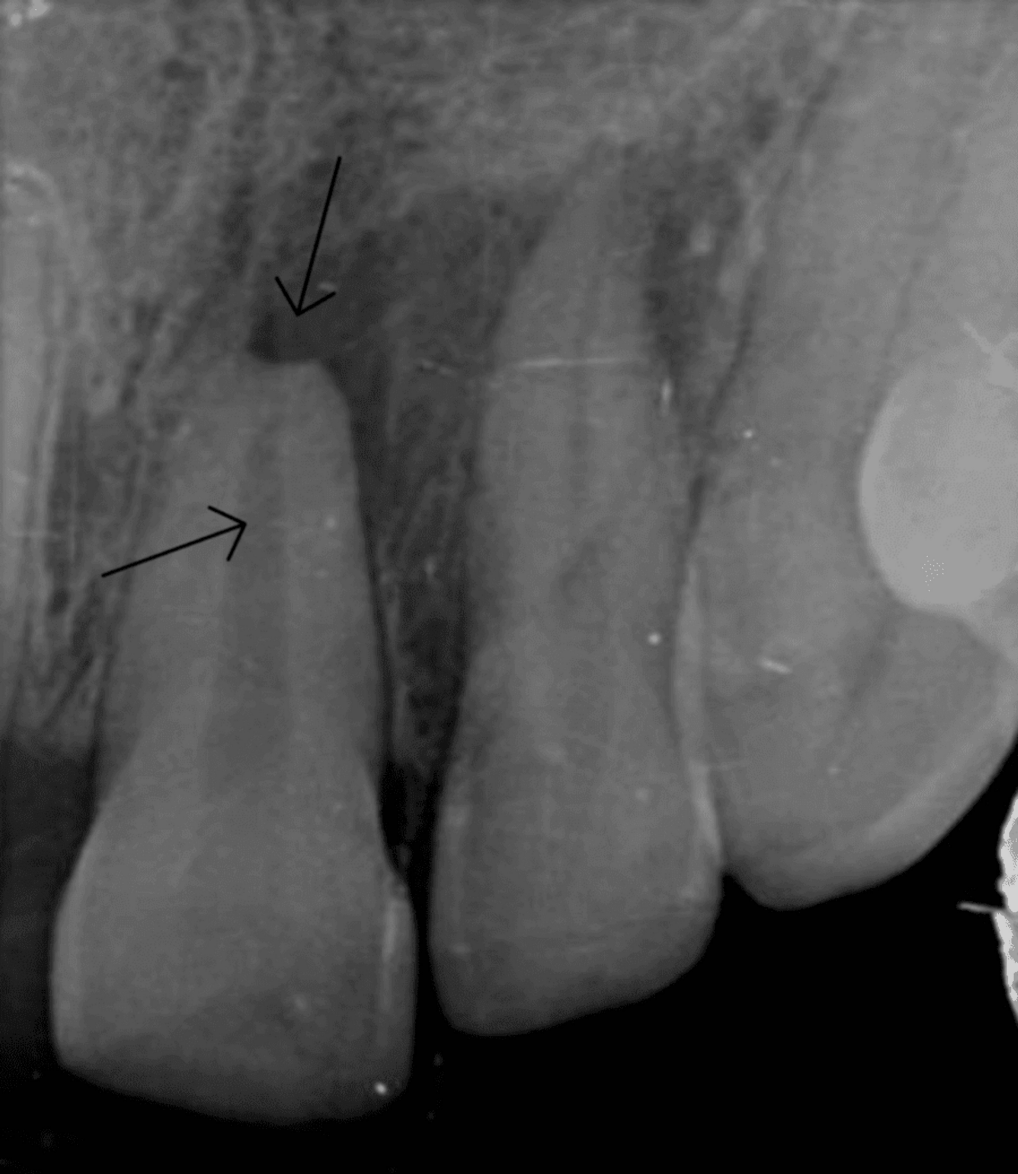
Preoperative intraoral periapical radiograph showing immature root with periapical radiolucency in tooth 21 (black arrows)

The cold test using refrigerant cold spray (Coltene/Whaledent, Switzerland) and electric pulp testing (Pulp Vitality Tester, Parkell, Edgewood, NY) revealed no reaction compared to the control teeth. An intraoral periapical radiograph indicated no reduction in the crown structure in tooth 21, underdeveloped roots in tooth 21 when compared to contralateral tooth 11, and radiolucency around the periapical region of 21 (Figure 2).

**Figure 3:**
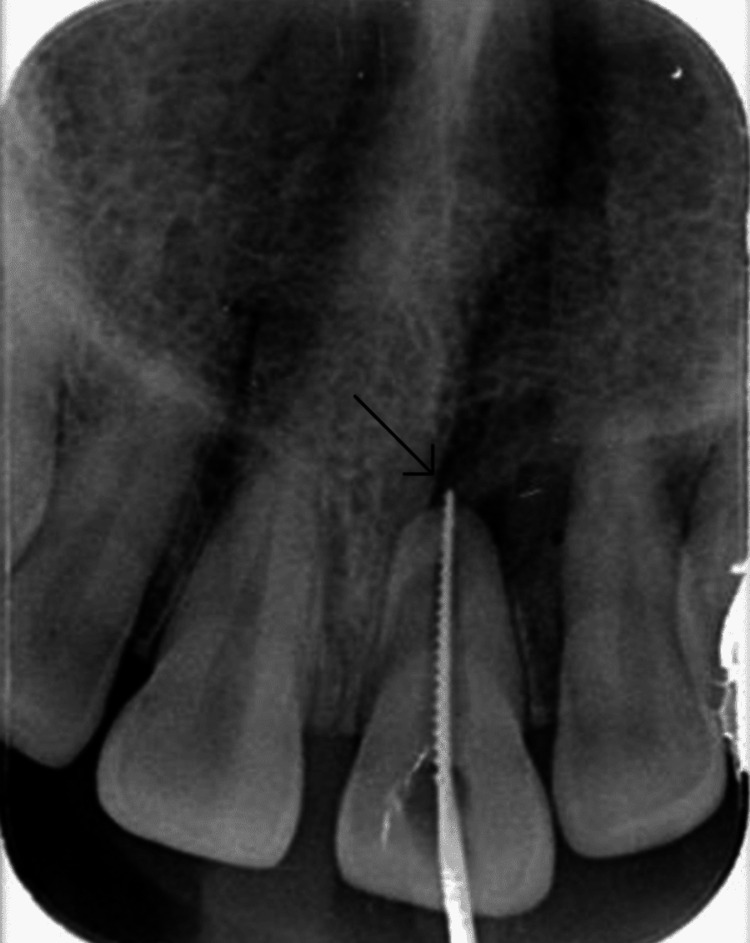
Intraoral periapical radiograph showing confirmation of working length determination (black arrow)

These data led to diagnosing pulpal necrosis and symptoms of apical periodontitis with evident periodontal ligament widening radiographically in tooth 21. The apexification protocol was applied for tooth 21. The tooth was isolated and prepared for magnification, and endodontic therapy was initiated (Figure 3).

**Figure 4:**
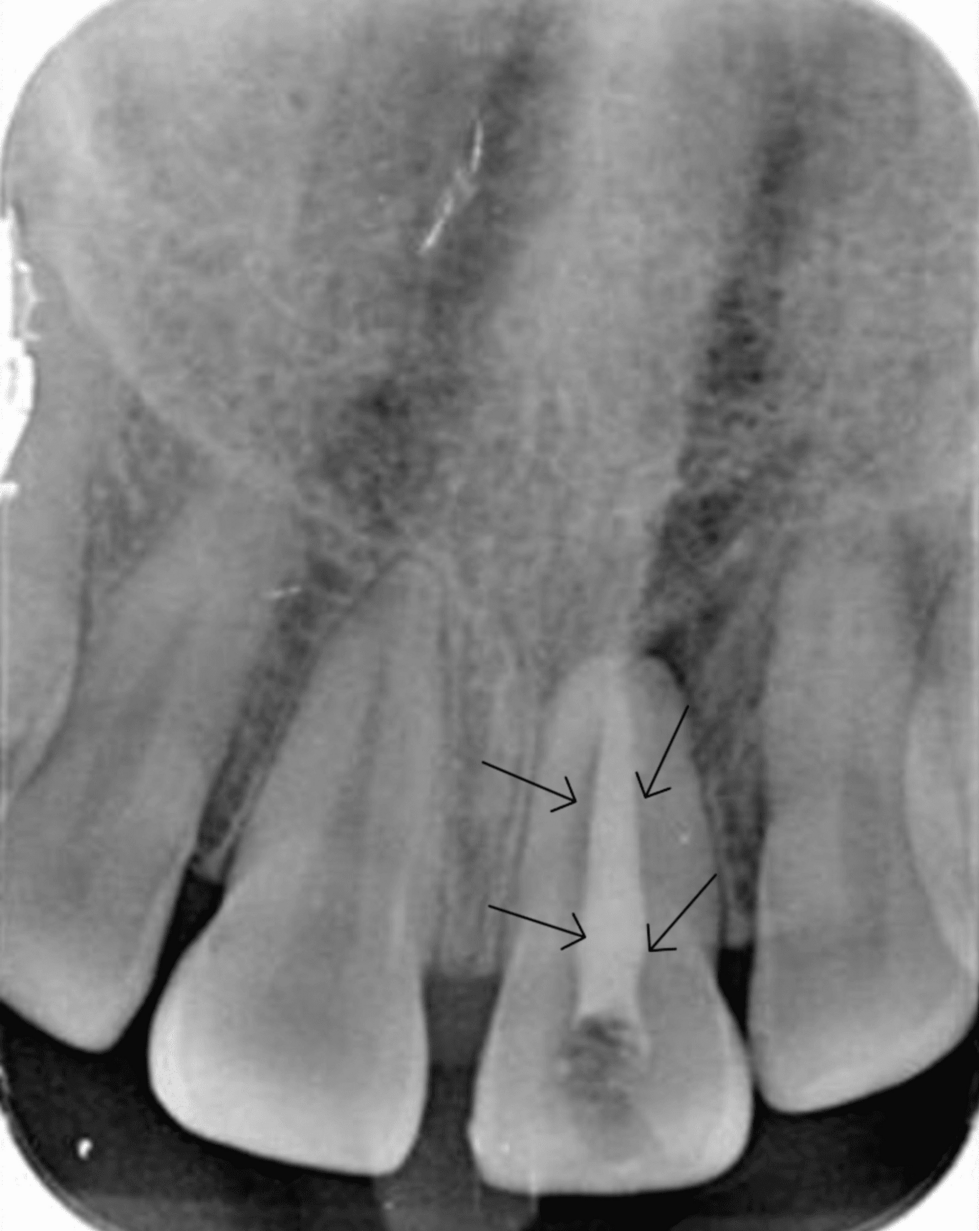
Intraoral periapical radiograph showing placement of intracanal calcium hydroxide dressing (black arrows)

During instrumentation, the apical end of the root was completely loose with no. 120 K file. Complete chemomechanical debridement with 20 mL of 3% sodium hypochlorite (NaOCl) was performed, and a calcium hydroxide dressing was placed for 10 days (Figure 4).

There were no reported adverse effects throughout the follow-up times. Once the patient returned without symptoms, the intracanal dressing was rinsed out with normal saline, final irrigation with a copious amount of 3% NaOCl, and flushed with 17% ethylenediaminetetraacetic acid.

**Figure 5:**
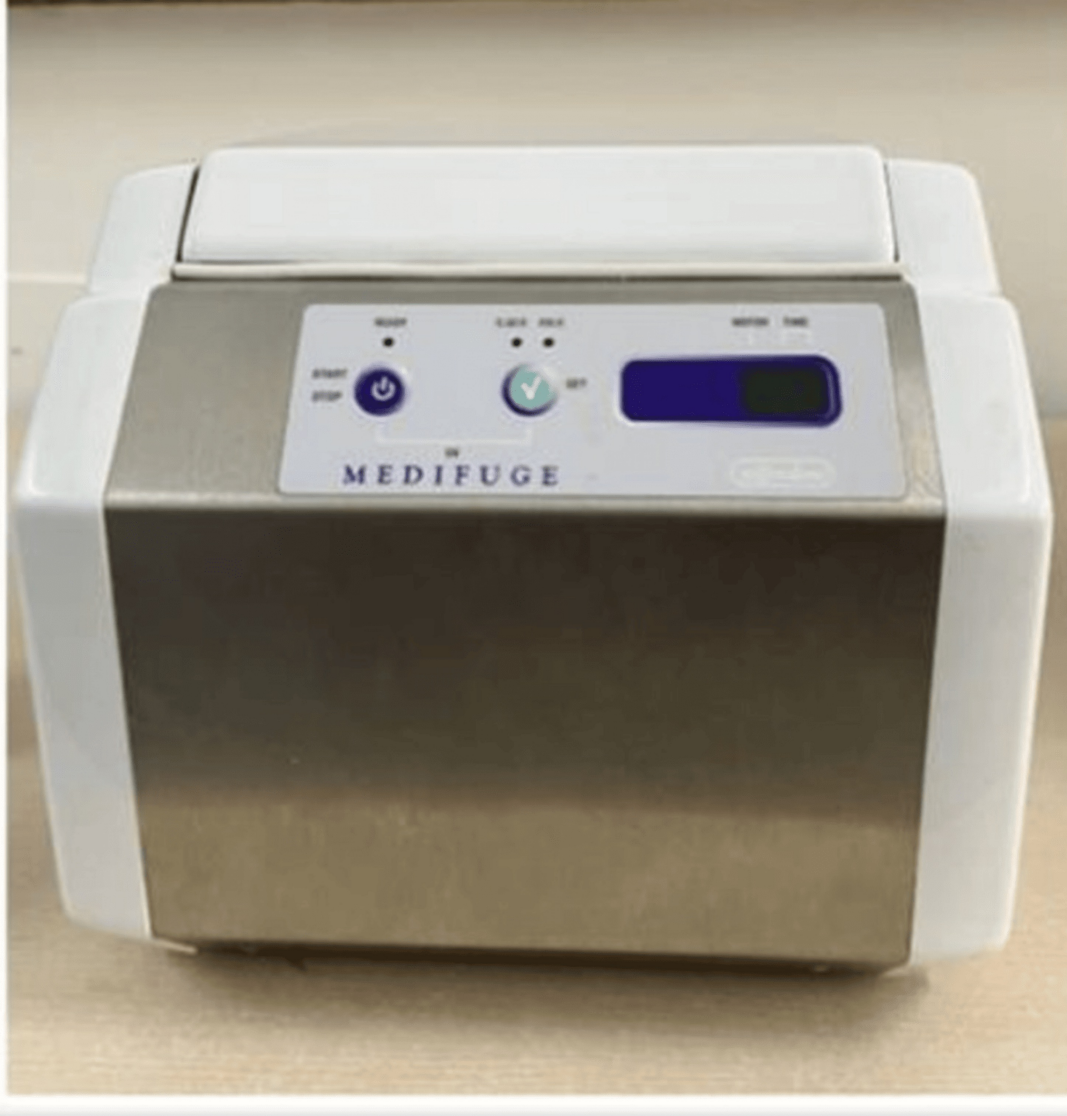
Medifuge centrifuge machine for CGF preparation CGF: concentrated growth factor

**Figure 6:**
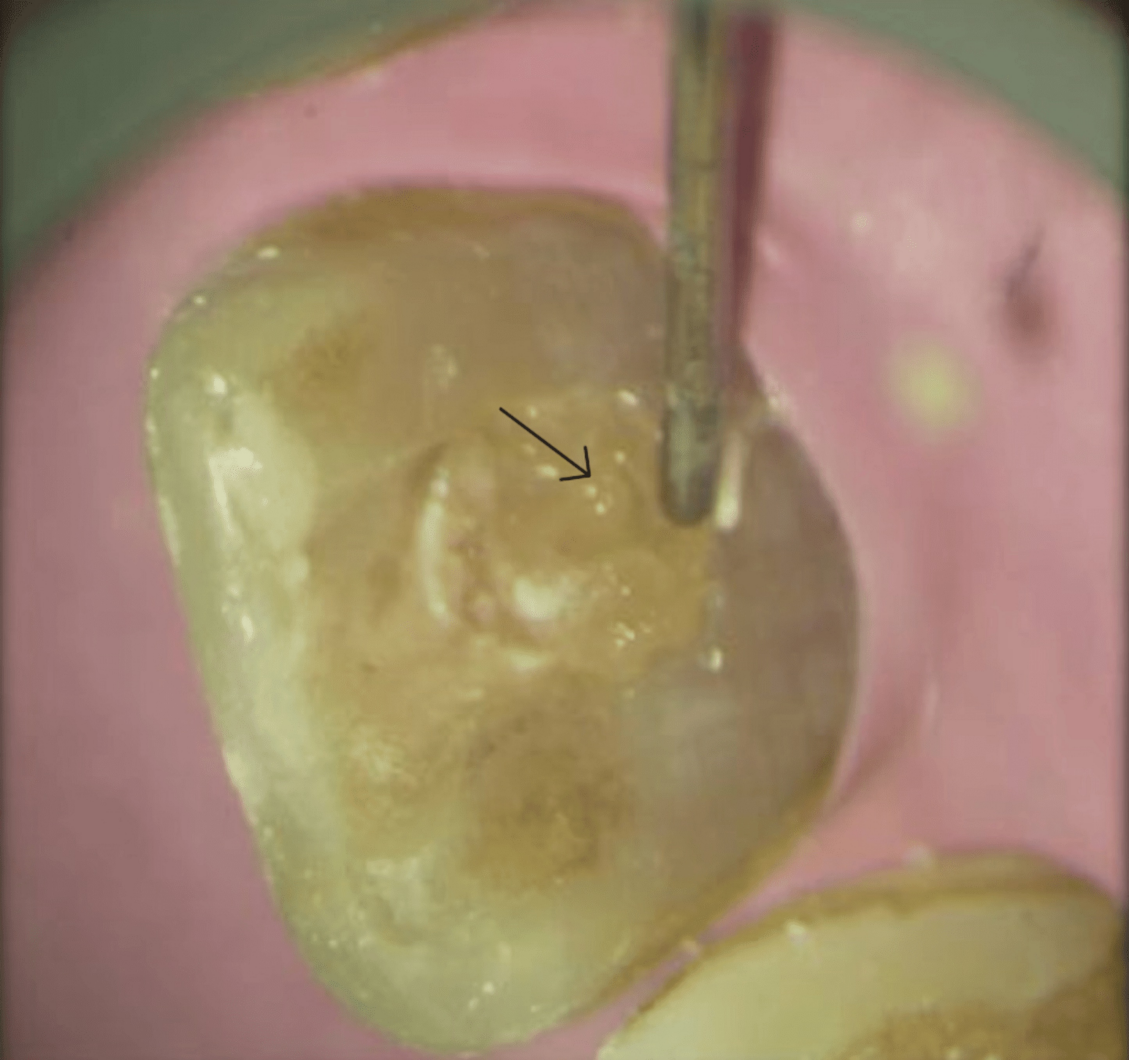
Intraoral magnification photograph showing delivery of the CGF (black arrow) CGF: concentrated growth factor

**Figure 7:**
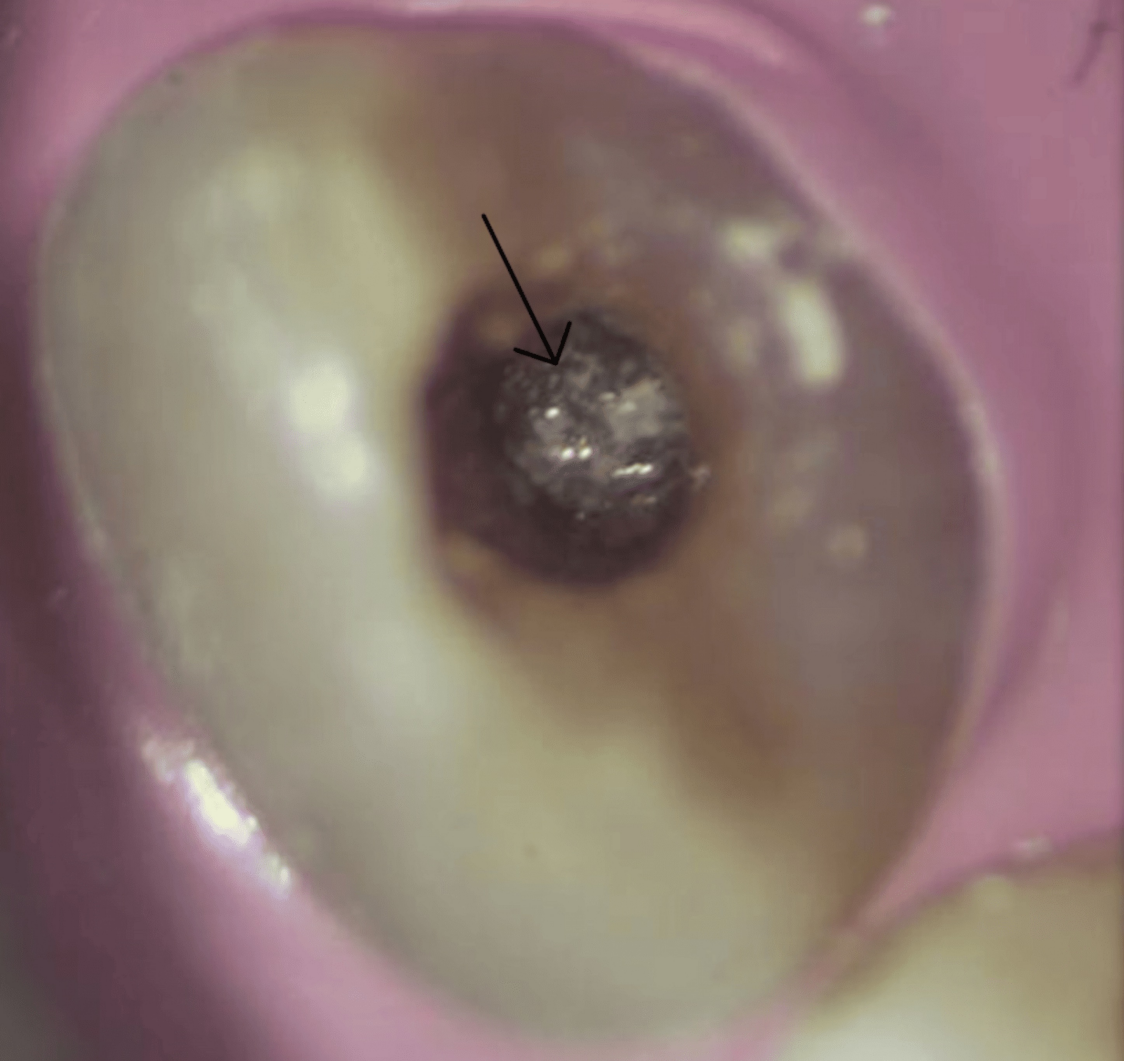
Intraoral magnification photograph showing exact placement at the apical end of the root canal

**Figure 8:**
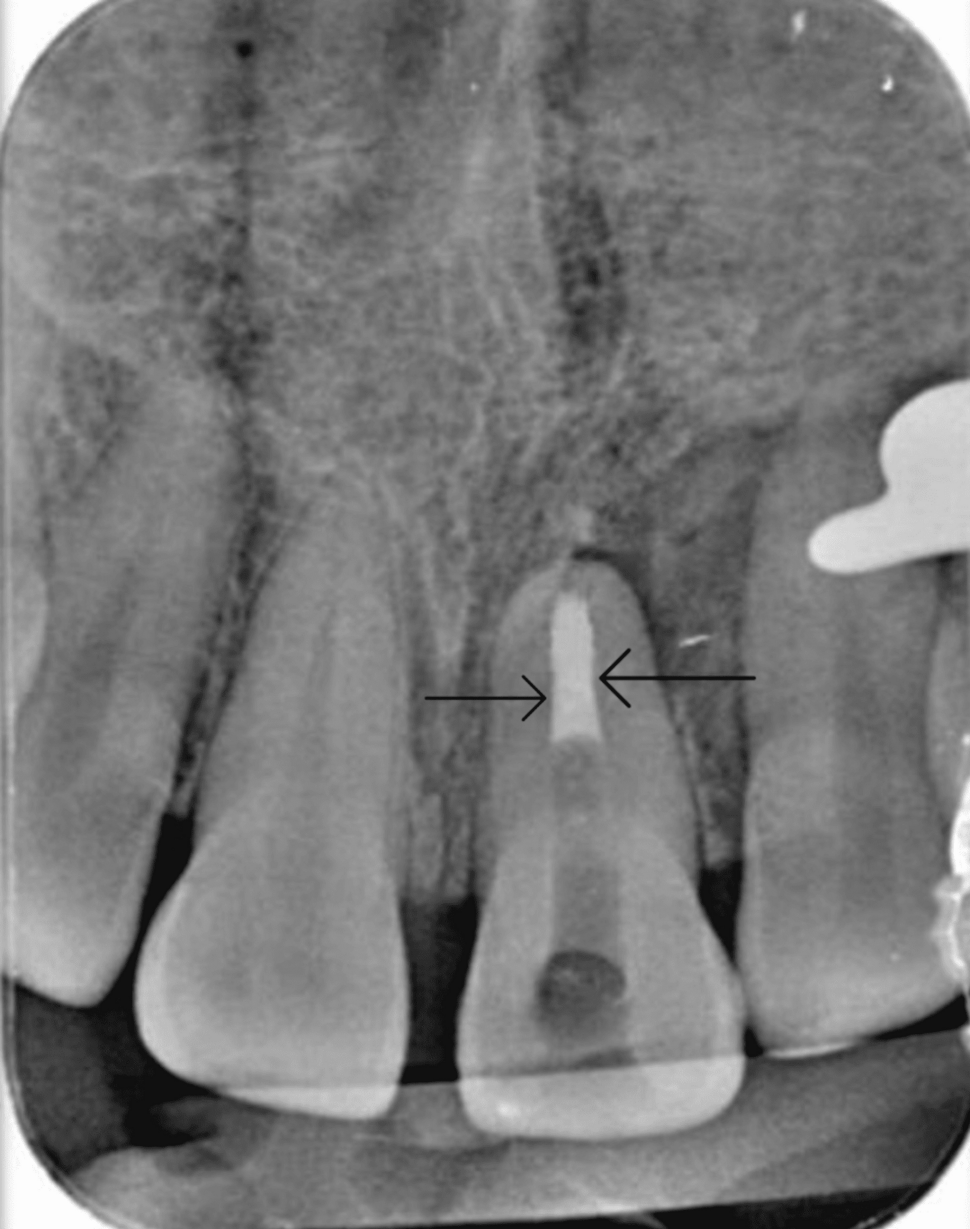
Placement of MTA confirmed with IOPA radiograph for the exact position and absence of voids (dense packing of MTA) (black arrows) MTA: mineral trioxide aggregate; IOPA: intraoral periapical

The CGF was prepared using a matched centrifuge apparatus (Medifuge, Silfradent SRL, Sofia, Italy) and two disposable 10 mL nonanticoagulant tubes. A 10-mL intravenous blood sample from the patient was obtained and put in anticoagulant-free centrifuge tubes. The centrifugation process included acceleration for 30 seconds, centrifugation at 2,700 rpm (two minutes), 2,400 rpm (four minutes), 2,700 rpm (four minutes), and 3,000 rpm (three minutes), followed by deceleration for 36 seconds to halt. Upon completion of the automatic preprogrammed cycle, the centrifugation tube held a mixture of four layers: red blood cells (RBCs) occupied the lowermost or fourth layer, growth factors were present in the third layer, which was made up of a fibrin buffy coat, and serum was present in the uppermost or first layer. The CGF-containing fibrin gel was removed from the RBCs. The resultant CGF was placed as a scaffold matrix to produce an apical matrix for MTA placement. After covering it with a 4-mm MTA plug (MTA Angelus, Brazil), the coronal cavity was filled with composite (Figures 5-8).

**Figure 9:**
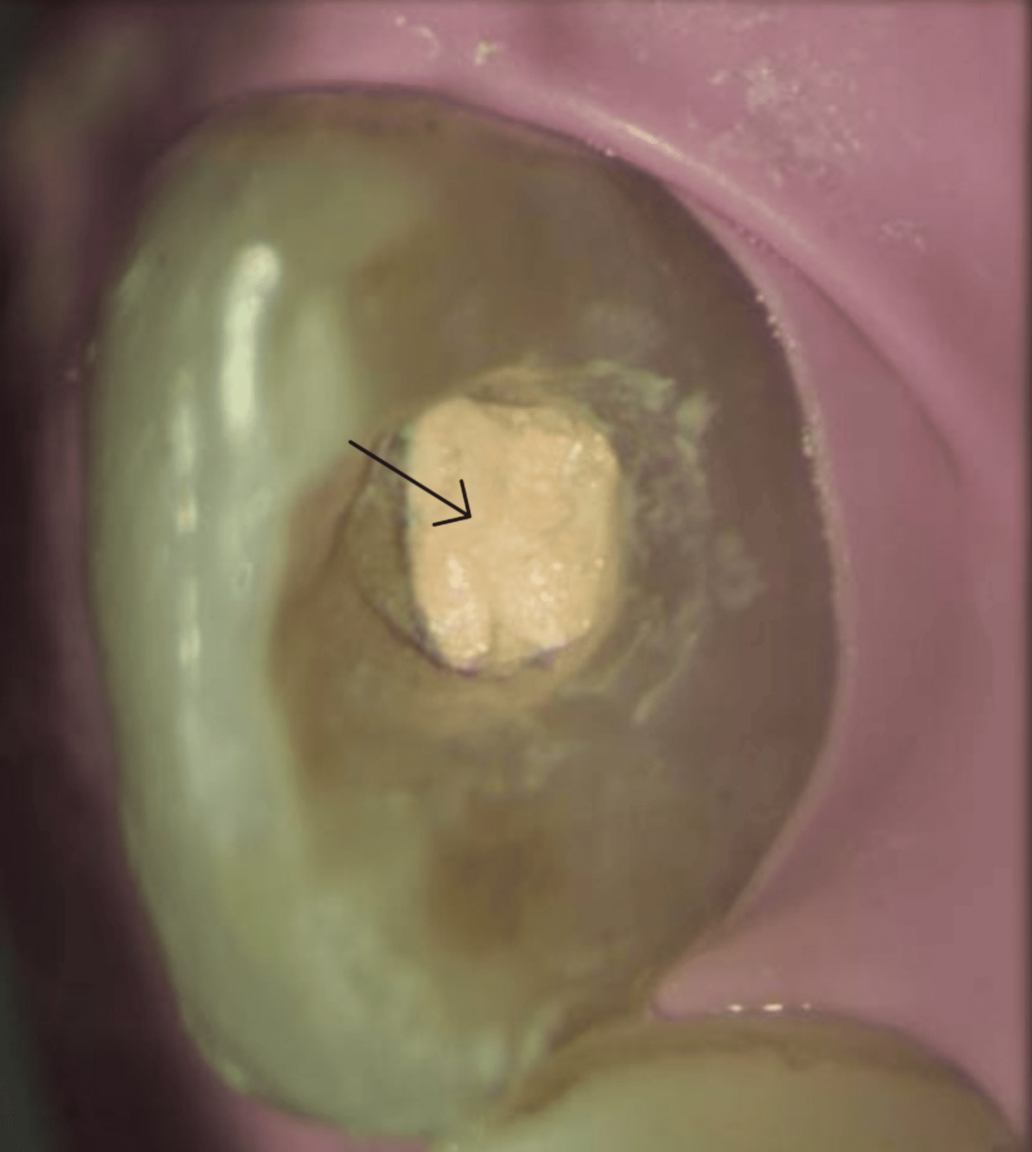
Clinical picture showing obturation (black arrow)

**Figure 10:**
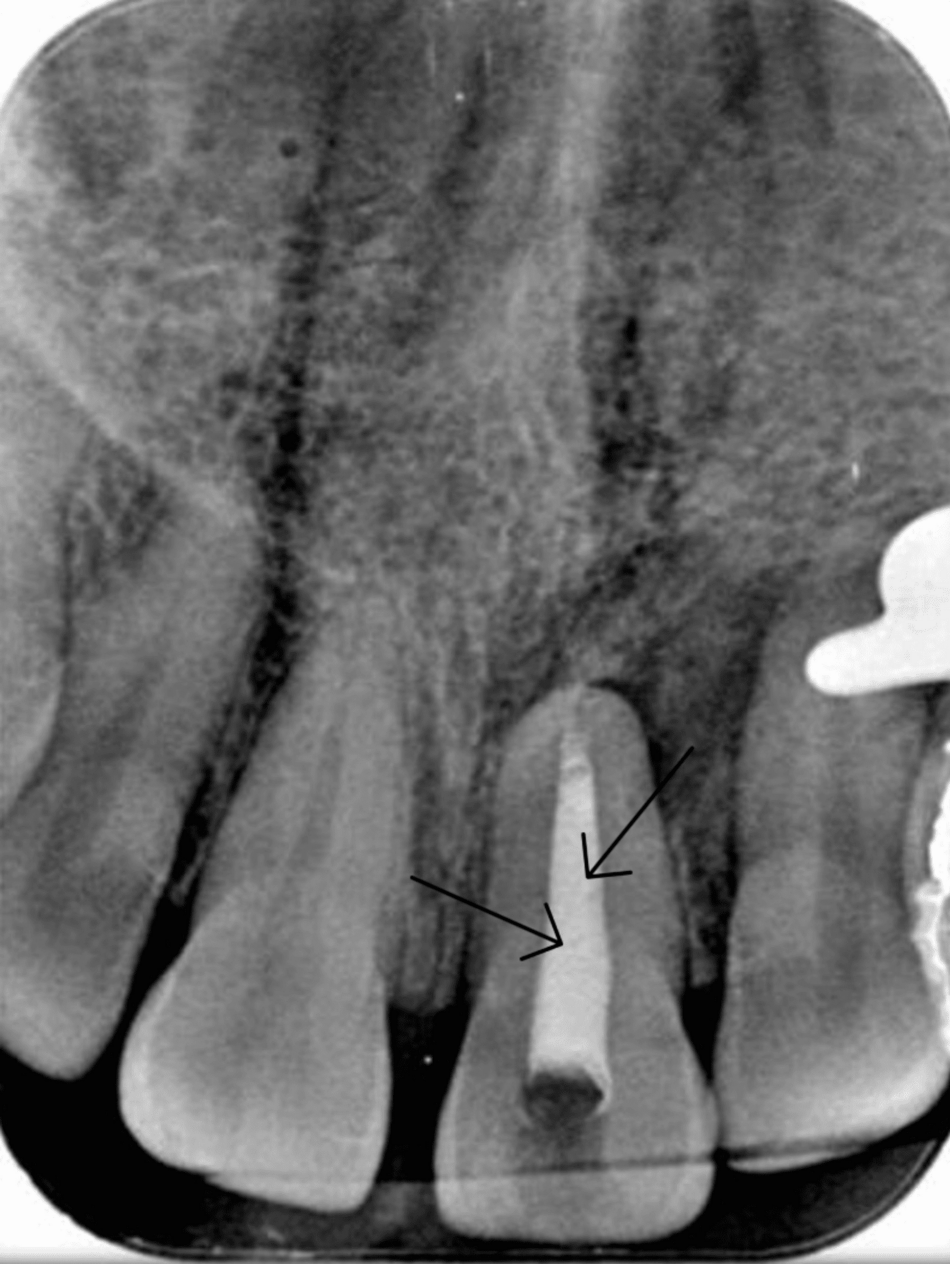
Immediate postobturation radiograph (black arrows)

The MTA (ProRoot MTA, Dentsply India Private Limited, Gurgaon, India) was sealed over the barrier (4 mm). The remaining canal was obturated using thermos-plasticized gutta-percha, and the access cavity was sealed. An immediate postoperative radiograph was taken (Figures 9, 10).

**Figure 11:**
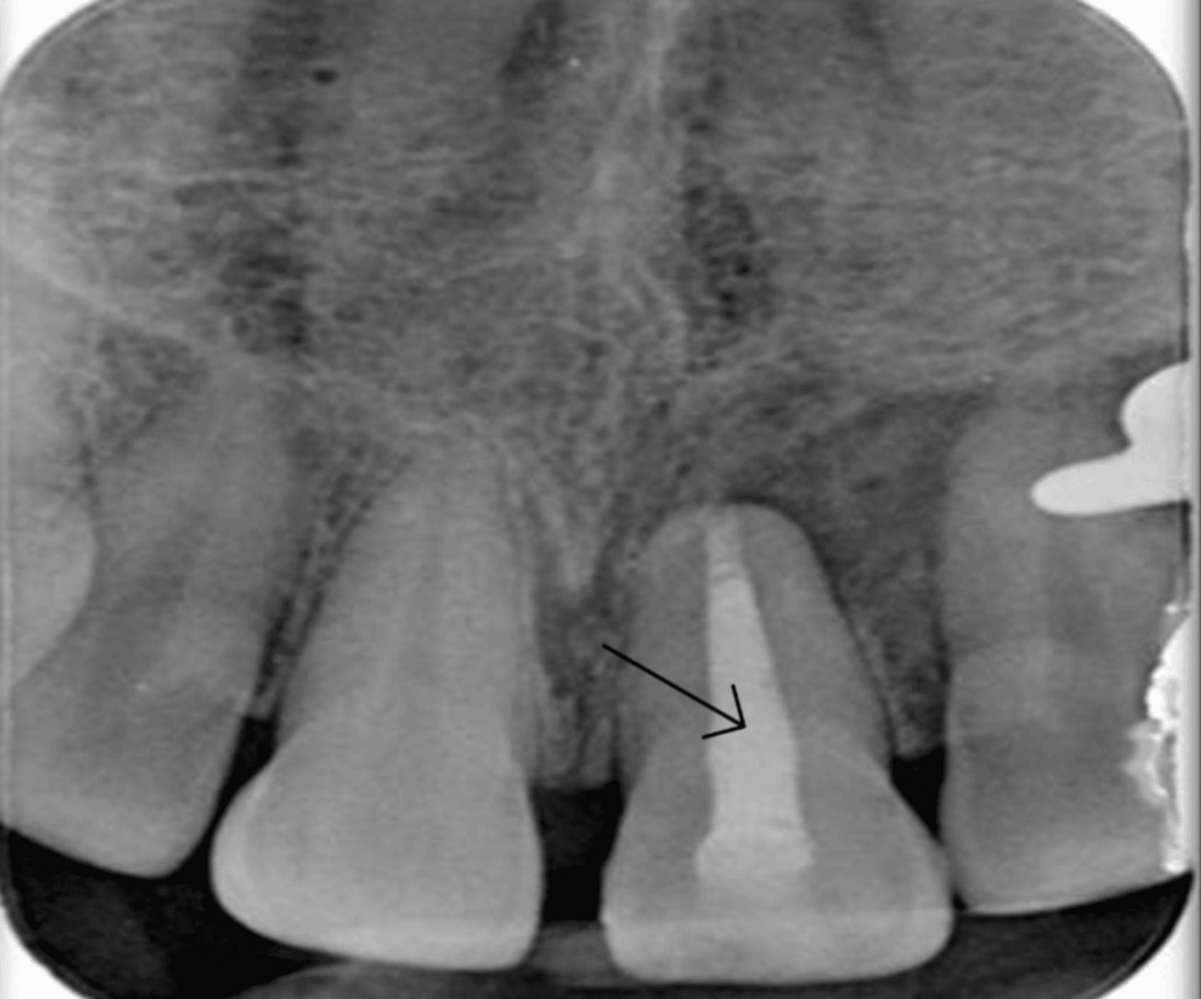
IOPA radiograph of the 12-month follow-up IOPA: intraoral periapical

A periapical radiography intraorally taken at the 12-month follow-up in tooth 21 revealed a decreased extent of the periapical lesion, progressing toward completion of healing with no clinical signs or symptoms (Figure 11).

## Discussion

Endodontic treatment for teeth with open apices and periapical lesions caused by trauma or abnormality includes traditional apexification with calcium hydroxide and contemporary MTA applications [9]. The main concern with a wide-open apex is the need to restrict the material to prevent excessive extrusion into the periodontal tissue [10]. Using a matrix prevents material extrusion into the periodontal tissues, eliminates leakage in the sealing material, and allows for a positive response from the periodontal tissues [11]. The apical barrier approach, which uses calcium sulfate or a mixture of calcium sulfate and collagen in powdered form, has been used previously. Various materials have been utilized to build the apical barrier during apexification [12].

One such apical barrier used as a matrix for apexification is based on biologically generated products from the host PRF. According to various case reports, the autologous platelet aggregate in the form of PRF has been used in the form for the management of open apex [13,14]. Second-generation platelet concentrations, such as PRF, are frequently used to expedite the repair of both soft and hard tissues by the release of their growth factors along with acting as apical barriers for placement of MTA. Its advantages over PRP are its low cost, lack of biochemical change, and ease of preparation and application.

In the present instance, an effort was made to use these growth factors to promote the repair of a tooth with open apices. The most recent generation of platelet concentrate products is CGF. Unlike PRP, CGF does not include bovine thrombin or anticoagulants, which might cause cross-infection and immunological rejection. It also releases more growth factors than PRP/PRF [15]. The present case report at 12 months follow-up showed no signs and symptoms clinically as well as radiographically.

## Conclusions

CGF has a great deal of potential to improve the repair and regenerative process using revascularization procedures to promote overall acceptable healing. In some cases, necrotic, infected immature teeth can be treated with total canal disinfection, and the autologous platelet aggregates may work well as a scaffold matrix in the treatment for open apex cases. This case report suggests that a combination of CGF and MTA can be a viable treatment option for teeth with radiolucent lesions and open apices, effectively repairing periapical lesions. The study's limitations include the requirement for long-term follow-up and a larger sample size to demonstrate CGF's efficacy.
